# A bioethics for all seasons

**DOI:** 10.1136/medethics-2014-102306

**Published:** 2015-01

**Authors:** Sarah Chan

**Keywords:** Ethics, Philosophical Ethics, Public Policy, Reproductive Medicine, Social Aspects

## Abstract

The last four decades have seen the emergence and flourishing of the field of bioethics and its incorporation into wide-ranging aspects of society, from the clinic or laboratory through to public policy and the media. Yet considerable debate still exists over what bioethics is and how it should be done. In this paper I consider the question of what makes good bioethics. Drawing on historical and contemporary examples, I suggest that bioethics encompasses multiple modes of responding to moral disagreement, and that an awareness of which mode is operational in a given context is essential to doing good bioethics.

## What is (good) bioethics?

Imagine, for a moment, being asked to compile a list of what you consider to be the top 10 works in the field of bioethics over the last four decades. Which books and papers spring to mind? If we were all asked to perform the same exercise, no doubt we would each come up with a different selection, though there might well be some overlap. But in trying to make such a list, we would undoubtedly have a sense of what it was we were looking for in our Bioethical Top 10. What, then, is that elusive ‘Oh! factor’ that makes good bioethics?

We might start by considering the influence of a given work in terms of its reach. Some journals produce a list of their most accessed or cited articles, presumably as some indication of the ‘best’ papers on offer. In this age of metrics and statistics, it is certainly tempting to fall back on numerical measures such as citation indices and impact factors—quantifiability as a proxy for quality. Then again, not all papers that are widely read or cited are necessarily good; notoriety does not always equal quality. Indeed, one might facetiously suggest that an effective way to be cited frequently is to be wrong! More seriously, a work that is open both to criticism and further development is of course likely to generate increased citations; the numbers, however, are but a crude reflection of this property and do not supply an explanation or description of what constitutes quality.

Perhaps, then, we ought to search for those works of bioethical scholarship that contain the most rigorous philosophical analysis, or the most elegant argument; or that provide insight into the most pressing issues of the day; or that enable the most effective policy solutions. But how do we measure any of these aspects, or decide which should predominate, in our determination of what we consider to be ‘good’? It is important to recognise that in asking what makes good bioethics we are also inviting an opinion about what we think bioethics is, or should be.

This is a problem that bioethics has confronted repeatedly throughout its relatively short history. In so doing, it has often turned a self-reflective eye to questions of disciplinarity and interdisciplinarity, method and methodology (or lack thereof)—in short, how to define what it is we do.^1^ Intertwined with such issues is the question of moral expertise, of what gives us as bioethicists legitimacy to carry out the roles we claim to be able to perform.^2^ ^3^

Bioethics has at least some of its roots in philosophy,^4^ but we do not, by and large, have an explicitly-developed ‘philosophy of bioethics’. Science produces explanations for observed phenomena and evaluates those explanations according to how well they describe and predict these phenomena. But if bioethics functions analogously, in attempting to elucidate some underlying moral principle, what are the phenomena we are trying to explain?

The role of moral intuitions in bioethics is a contested one, and being led by the ‘moral nose’ is often looked upon as being a poor method of ‘doing bioethics’. Yet bioethical reasoning often relies first on appeals to intuition to establish the basic premises from which axioms can be abstracted and against which more complex problems can be compared and analysed. Can we claim, then, that we are uncovering some sort of ‘moral truth’? This problem both recapitulates perpetual meta-ethical debates over the nature of moral philosophy itself and invokes issues of moral expertise—who is best placed to know or discover the truth, and whose statements should be considered as good moral guidance? How do we decide what makes something a ‘good’ moral explanation, and is producing moral explanations the proper purpose of bioethics?

Considering, almost a decade ago, the question of what bioethicists do and what claim to authority they have in doing it, Baker wrote: “the role of the bioethicist is not that of watchdog, policing and protecting the boundaries of morality, but rather of facilitator, assisting society to reflectively articulate, interpret, and specify our common morality in the context of the rapidly evolving world of biomedicine.”^5^ What happens, though, when there is a lack of consensus on a single ‘common morality’? In fact these are the problems in which bioethics is most often called to take a hand—if there were no disagreement over what constituted ‘common morality’, there would be no disagreement over what should be done. The demand for bioethics, then—our justification for existence—can be seen as being generated by the need to respond to a lack of moral consensus within society. The measure of good bioethics on this account is the degree to which the response serves the need. But what form should our responses take and what is their function?

## Theory and practice: different bioethical hats?

Presumably we need to have an account of what bioethics is or does, in order to say what constitutes *good* bioethics, but this does not mean there must be a single account of bioethics or that it must be rigidly or precisely defined. Bioethics can be many things, depending on the particular context and purpose of the endeavour. A primary feature of ‘good’ bioethics, therefore, is ‘using the right tool for the job’. This in turn entails knowing what the job is we are trying to do; knowing (to mix metaphors) which hat we are wearing at any given time. To whom are we talking and for what purpose?

Bioethicists sometimes see themselves as divided up into schools of thought characterised by a particular method or approach. From a more general perspective, however, we might argue that the most significant divide in bioethics is not between virtue ethics and consequentialism, or continental and Anglo-American philosophy, but between theory and practice: bioethics-as-philosophy versus bioethics-as-policy.

Bioethics is frequently described as a branch of practical ethics or applied philosophy. A common bone of contention between differing accounts of bioethics, however, is the extent to which a given piece of work needs to have practical application in order to have value or ‘count’ as bioethics. It is often asserted that bioethics must be ‘action-guiding’, should tell us what to do. Some, indeed, see bioethics as defined by this aim: Sheehan and Dunn, for example, assert that bioethics “must primarily be concerned to address a *practical ‘ought’ question*”.^6^

In judging what is a worthwhile piece of bioethics, though, we should caution against a too-literal interpretation of practical applicability. An argument does not always have to translate directly into policy or have an immediate and measurable real-world outcome in order to be of some practical value; the ways of moving towards answers to “practical ‘ought’ questions” are not always themselves practical. Consider some of the best known thought experiments generated by bioethics in its early years, for example, involving trolleys and violinists and kittens. Needless to say, such thought experiments do not and are not intended to represent actual situations that are ever going to occur in real life! Moreover, it is worth noting that bioethics does not produce a single ‘solution’ to such problems. The aim of the exercise is not to solve the problem as such, to know what to do should we be confronted with such a situation, but to provide some sort of structure to how we think about the much more complex, messy, squishy problems we encounter in real life—to abstract certain dimensions of these problems and render them susceptible of analysis.

Likewise, some of the terrain into which bioethics has ventured in more recent years has attracted criticism for being too speculative. To give a personal example, when discussing more radical forms of human enhancement, such as living to hundreds of years old or splicing our bodies with machines, I have at least once been asked “Why are you talking about this? This is science fiction! Why aren't we discussing saving the rainforests?” While not discounting the importance of the rainforests, I fervently maintain—and the existence of an ever-growing body of literature on the topic suggests I am not alone in holding this opinion—that an inquiry into the ethics of cyber-enhancement, for example, humans becoming cyborgs, or uploading our brains to computers, can be a worthwhile bioethical enterprise. Its worth, however, is more in what we learn about our present selves and values than in addressing ‘practical ought questions’ about whether we should adopt a technology that does not yet exist.

This is not to say that such pursuits are purely blue-skies bioethics, producing only pure moral knowledge that lacks all practical application (indeed, depending on one's view of how bioethics works to generate moral knowledge, one might deny that such a thing as ‘pure moral knowledge’ can even exist). Using the above example, the philosophical understandings of the ‘human body’ concept and its associated values that we gain through bioethical imaginings of cyber-enhancement can also inform our consideration of more timely ‘ought’ questions regarding current forms of embodiment and the normativities that attach to them, such as disabled/moderately enhanced bodies and their relationships to assistive technologies. Or, to take another example, contemplating how we would or should treat a chimeric mouse or chimpanzee with human-like intelligence—unlikely as it may be that such a being will soon be created—may reveal moral insights that bear upon how we ought to treat non-chimeric mice, chimpanzees and other animals in the present day.

Critics who accuse us of being unrealistic with reference to the thought experiments we invoke, or of dabbling in science fiction with reference to the technological possibilities we imagine, are therefore missing the point. The value of conducting thought experiments that are themselves unrealistic and speculating about technological developments which are as yet only futuristic possibilities lies not least (though also, I would venture to suggest, not only) in what such considerations can tell us about real and present bioethical problems.

So, doing good bioethics can sometimes entail letting our bioethical imaginations run wild. Nevertheless, intersection with the real world remains an important dimension of (and justification for) bioethics, demanding that the work we do be relevant, understandable and applicable, at least when it is offered as a response to a real-world dilemma. This, however, leads us to another potential quagmire of criticism or crisis of bioethical identity: that bioethics is not good philosophy, or that only good philosophy can be called bioethics.

## Too philosophical, or not philosophical enough?

Where our aim as bioethicists is to influence policy, it is crucial not only that our arguments are adequate and appropriate to the task but also that they are presented in a manner that will be effective. But what to do when effective presentation involves glossing the underlying philosophy, or when the argument is philosophically sound but contextually or socially inappropriate? The problem of how to walk the line maintaining both philosophical scholarly integrity and the public acceptability necessary to retain credible influence has been a long recognised tension in bioethics.

We may think that good bioethics should aim at “actually convincing people to act differently or to change policy because of the arguments and answers that the bioethicist provides”,^6^ but this says nothing about how these arguments and answers should be derived. If this is indeed what we think our job is, then privately reasoning our way to what is morally right and publicly presenting views aimed at changing policy or behaviour to achieve a morally better outcome are two linked but distinct faces of bioethics.

This can be illustrated by reference to two examples from across the lifetime of what we might call contemporary bioethics in this country: the seminal report of the Warnock Committee on embryo research in 1984 that eventually led to the foundation of the Human Fertilisation and Embryology Authority (HFEA), and the policy recommendations produced by the HFEA's own consultation on mitochondrial replacement, 30 years later.

The activities of the Warnock Committee and the resulting Warnock Report have been widely seen as a test case for the role of philosophical bioethics in policymaking. Commentaries at the time criticised the approach and reasoning by which the committee arrived at its recommendations, notably whether the commitment to compromise was an appropriate response to moral pluralism, and whether there was any sound moral basis for selecting the 14-day limit as the ‘bright line’ after which research should be prohibited. But producing watertight philosophical reasoning and dispensing moral expertise was not the committee's self-stated mission. Instead, as Warnock herself argued, the aim was to facilitate a dialogue and process by which the plurality of dissenting moral views could somehow forge a workable policy decision.^7^

Thus, as Nelson comments, the choice of the 14-day cut-off “…did not reflect an overwhelming national feeling that individuation is an essential property of a member of the human species. On the contrary, utilitarian considerations concerning potential research benefits played an explicit role in the Committee's judgment about this matter.”^8^ The fortuitous intersection of scientific utility (most useful research could be done before this stage), the attributes perceived as important by much of the public, and the use of individuation as a superficially plausible property to support the cut-off point enabled a compromise position on this most intractable issue. While the description of the Warnock Committee report as containing ‘more or less well defended views’ may seem to be damning with faint praise, in fact it was the characterisation of bioethics as a policy process, in the work of the committee itself and in its key recommendations for oversight (eventually taking form as the HFEA), that led to a legacy of what has been for the most part effective public policymaking over the 30 years since, while many other jurisdictions were still struggling to move past moral disagreement and lacking functional policy on the matter.

The work of the Warnock Committee, then, is undeniably one of the most influential pieces of British bioethics, both in its effects on policy and research and in defining what bioethics itself has today become. It may not have been the most philosophically consistent work of all time, but was it ‘good bioethics’? Considering all of the above, I think the answer is yes.

Moving forward to the present day, the work of the HFEA and other policy groups on mitochondrial replacement^9^ ^10^ provides another example of favouring the politic over the philosophical to achieve the best policy outcome. Much of the controversy over this technique centred on the significance of genetics, and whether the 0.1% of DNA contained within mitochondria would affect either the child's parentage or identity. The issues of genetic parentage and identity both offer considerable scope for philosophical and conceptual analysis—what makes a parent?; what constitutes identity?—but although such analysis may deepen our understanding of these complex concepts, it is perhaps not immediately useful in a policy-setting context where concerns over ‘three-parent babies’ dominate the media coverage of the subject.

The consultation reports, then, downplayed the significance of mitochondrial DNA in genetic kinship ties and identity, emphasising that it contributed very little to the child's genetic make-up and would not affect any essential characteristics. The latter claim is clearly over-reaching: mitochondrial DNA affects at least one important characteristic, that of health status; otherwise the technique would be pointless. And although a careful consideration of the meaning of ‘parenthood’ might well conclude that mitochondrial donors should not be considered parents, the three-parent issue was sidelined rather than analysed.

At a public meeting to discuss the HFEA consultation in March 2013, comments made by those involved suggest that the de-emphasisation of the importance of genetics to identity was strategic. Genetic parenthood and mtDNA were deliberately dissociated because the three-parent label was seen as too controversial to be publicly acceptable, and the decision to favour donor anonymity and not to regard donors as genetic parents was influenced by fears that the technique might be ‘jeopardised by a three-parent tag’ (D Griffiths 2014, unpublished data).

While this is not the place for an exposition of the moral arguments supporting the use of mitochondrial replacement, such arguments do exist and are in my opinion well-founded. I do not doubt that permission, rather than prohibition, is the morally better policy in this case. The consultation, however, did not take the route of analysing and exploring these arguments philosophically; its recommendations were arrived at by a different but arguably more effective means. If on the basis of this, the technique is allowed to proceed, the better outcome will have been achieved.

I want to suggest, then, that at least in the policy context, it is sometimes less important to be absolutely precise or ‘correct’ (or truthful, if we think there is such a thing as moral truth) in our arguments than to be convincing in order to achieve the desired outcome. If what we care about are consequences, then, in some cases, the means of less than perfect moral reasoning may be justified by the ends of a functional policy that achieves the best outcome. Granted, we must have some underlying idea of what is ‘right’ in order to decide what policy outcome we want to achieve; as bioethicists in whatever capacity, we are not obliged, nor perhaps is it possible, to maintain a fiction of moral neutrality. But the mechanism for achieving the desired outcome does not always have to correspond perfectly to our reasons for thinking it is desirable.

## What do we care what other people think?^i^

In more recent years, another point of contention within bioethics has been the ‘empirical turn’ and (perhaps overstated) disciplinary rivalry between philosophically and sociologically-based approaches to the contested terrain of understanding the effects of biomedical technology on society. This is sometimes conceptualised as a conflict between competing approaches, the normative versus the descriptive. But to see these as mutually exclusive perspectives in the process of doing bioethics is, again, to take too narrow a view of what bioethics itself is and does.

Consider, within the mitochondrial replacement debate discussed above and in reproductive ethics in general, the contested concepts of ‘family’ and ‘parenthood’. An analysis of these may be valuable in addressing many contemporary ethical problems in reproductive medicine, but it must be remembered that these concepts are themselves social constructs; a bioethical analysis of ‘family’ is meaningless without a socially-contextualised account of the concept, and the application and applicability of the results is likewise socially contingent. Sociology is necessary and integral to the bioethical process as a whole, even if isolated elements of our research do not draw directly upon it.

A similar perspective can be applied to the role of public consultations in bioethics. Some scholars scoff: why should we turn to the general public for their ‘inexpert’ opinions regarding matters on which we supposedly have more authority? (Again, the disputed issue of moral expertise arises here). But this mistakes the function of such exercises. The aim of public consultations is (at the risk of drawing a false distinction between ‘bioethicists’ and ‘everyone else’) to tell us, not what ‘we’ should think but, at least to some extent, what ‘they’ think.

One advantage of this might be that we can better target and hone our arguments to serve their intended purpose, but again, to see this as the main or sole aim of public consultations risks caricature or over-simplification. Public engagement in relation to bioethics is not just finding out what ‘the public’ think so we can work out how to convince them of what is right, because we know best; the engagement process is itself important in validating policy decisions. We may perhaps draw an analogy here with the shift from public understanding of science to public engagement—the aim of the exercise is not simply to enlighten the public with our superior scientific or moral knowledge, but to engage the public in dialogue via which science and bioethics, both being social enterprises, can proceed.

This again implies that, in the context of public engagement over ethically contested policy decisions, it is less important to be right or to express a perfectly consistent moral position than it is to arrive, via *this process*, at a workable solution. This is aptly illustrated by the examples above: policy-oriented bioethics is not identical to philosophical bioethics. It is true that we use apparent moral inconsistencies in policy as a way back into the philosophical debate, to start picking apart moral arguments—a classic example of this is comparing policies on abortion and embryo research and their implications for the moral status of the unborn. But while this analysis goes on, a functional (even if not fully consistent) policy compromise is in place.

## Bioethics as negotiation: should we be careful what we say?

A brief diversion: what I have so far said focuses largely on bioethics in the UK context, when in fact the original instruction for this piece was to write about good *medical* ethics in general. Not wanting to become embroiled in a terminological debate (this is the *Journal of Medical Ethics* but many of the papers published herein would fall as easily under the heading of ‘bioethics’, were we to attempt to draw a distinction), I simply re-interpreted the theme, but it is perhaps worth taking a step back to comment on one aspect of a potential bio/medical ethics divide. The role of bioethics as public discourse has emerged particularly in the UK, but across the Atlantic, medical ethics has developed as an increasingly professionalised field of expertise, with clinical ethicists appointed to specialist positions within hospitals.

The activities carried out in the capacity of professional clinical ethicist are often very far from the role of the philosophical bioethicist, leading deVries to comment that “[c]linical bioethicists in the US seem to be doing a form of social work or dispute resolution”.^11^ Nonetheless, this may also be a valid response to moral conflict, the ethicist's role here being to do with shared or delegated responsibility and accountability for actions between parties and within organisations. This provides us with another potential way of understanding bioethics: clinical ethicists negotiate the personal relationship between private individuals and healthcare organisations; bioethicists negotiate the relationship between the policymakers of the state and the citizens of its publics.

The role of a good negotiator, then, requires attention to diplomacy. Richard Ashcroft describes the role of the bioethicist as that of a ‘public intellectual’;^12^ as public intellectuals, we have a responsibility to be aware that our words will be heard by the public and to consider how they may be interpreted. This is another aspect of the ‘multiple-hat problem’ of the bioethicist's differing roles: knowing when and how to exercise discretion over what we say. Again, writing about the Warnock report and whether it represented a bioethical compromise or compromised bioethics, Benjamin suggests that bioethics-as-philosophy should take the ‘no holds barred’ approach “with little or no regard as to immediate acceptability… little or no concern for short-run effects on popular sensibilities”^13^ but goes on to acknowledge that bioethicists can play a different role in policy, so long as the distinction is clearly understood and observed.

This may be obvious but bears repeating, given the potential for loss of public trust and consequent impairment to the effective functioning of bioethics should a message drafted while wearing one hat be delivered under another. (Readers of this journal, of course, witnessed a recent unfortunate example of this with the public reception of the idea of ‘after-birth abortion’:^14^ ^15^ sound words from philosophers, perhaps rash ones from public intellectuals.) Doing bioethics requires an awareness of the multiple roles that bioethics is called upon to play, and knowing how to balance them is a part of good bioethics.

## New horizons: to the next 40 years

Going back, in conclusion, to our hypothetical ‘Bioethical Top 10’, we are perhaps no closer to agreeing on the specific membership of the list, but can say some things about its general content. Good bioethics is likely to have been produced in response to moral disagreement of some sort; lack of moral consensus, rather than presenting an insurmountable obstacle, provides opportunity for discourse and generating new understandings. These understandings may range from theoretical to practical; all are valid spheres for bioethics to operate within, so long as we are aware of what our purpose is in generating them, and conscious of how we present them and to what end. The ‘Oh! factor’ in bioethics comes from something that allows us to approach or understand a problem in a new way; that provides some new insight into the nature of a problem, its intersection with social or regulatory aspects, a concept itself or how that concept is deployed; that moves, however propelled, towards a better world.

In so doing, bioethics achieves one further thing: it opens up space for further reflection and inquiry. It does not have to, nor should we expect it to, provide the definitive solution to all moral problems. If such a Grand Unified Theory of bioethics were possible, the field would be closed rather than open-ended; we would one day know everything there was to know about morality and no more thinking would be possible; and that would be a sorry day. Good bioethics breeds more bioethics. Long may it continue!

## Footnotes

i Without apologies to Richard Feynman.
